# Gut microbiota alliance to shape sceneries of familial Mediterranean fever: a scoping review detailing difference between children and adults

**DOI:** 10.3389/fimmu.2026.1814103

**Published:** 2026-03-18

**Authors:** Camilla Maria Pisa, Angelo Verrone, Martha Mazuy, Lorenzo Ientile, Donato Rigante, Susanna Esposito

**Affiliations:** 1 Pediatric Clinic, University Hospital, Department of Medicine and Surgery, University of Parma, Parma, Italy; 2 Department of Life Sciences and Public Health, Fondazione Policlinico Universitario A. Gemelli IRCCS, Rome, Italy; 3 Periodic Fevers Research Center, Università Cattolica Sacro Cuore, Rome, Italy

**Keywords:** autoinflammatory disease, dysbiosis, familial Mediterranean fever, gut microbiota, inflammasome, innate immunity

## Abstract

Familial Mediterranean fever (FMF) is the most common monogenic autoinflammatory disease worldwide and a key-model to illustrate dysregulation of innate immunity, etiologically determined by pathogenic variants in the *MEFV* gene, encoding pyrin, leading to uncontrolled interleukin-1β and interleukin-18 release. Despite its genetic basis, FMF shows marked clinical heterogeneity in all-aged patients, mostly in children, suggesting a role of potential environmental modifiers which are far to be exactly unraveled. Recent medical literature has increasingly illuminated the importance of gut microbiota in maintaining overall health and immune functions, and its contribution has been claimed also to explain both FMF inflammatory activity and heterogeneous disease expression. This narrative review summarizes current evidence on the interaction between gut microbiota and FMF, with a specific focus on differences between children and adults. Pediatric studies dedicated to FMF have reported intestinal dysbiosis in terms of reduced microbial diversity and depletion of short-chain fatty acid-producing bacteria, with subsequent enrichment of pro-inflammatory taxa: such alteration could modulate pyrin-inflammasome activation and contribute to systemic inflammation, disease phenotype, and response to colchicine or to other drugs specifically used in colchicine-resistant FMF. Geographic and lifestyle factors may shape intestinal microbiota composition early in life, reinforcing the relevance of gut flora and confirming its activity as a crucial tessera to determine FMF sceneries, mostly in children, and a potential target for future add-on therapeutic strategies. In addition, colchicine therapy appears to partially remodel the gut microbiome, empowering a local beneficial anti-inflammatory microbial profile.

## 1 Background

The most frequent and best-known monogenic autoinflammatory disorder across all continents is familial Mediterranean fever (FMF), caused by biallelic mutations in the *MEFV* gene, which regularly encodes a cytosolic pattern-recognition receptor, named *pyrin*, expressed in neutrophils, monocytes, dendritic cells, and synovial fibroblasts: the disease is heralded by recurrent fever attacks and serositis, representing an archetype to investigate specific causes of dysregulated innate immunity (1). On physiological conditions, pyrin is tightly regulated by a complex phosphorylation process and strict binding to regulatory proteins, keeping the pyrin-inflammasome at a quiescent state. However, FMF-associated *MEFV* pathogenic variants impair this inhibitory mechanism, lowering the threshold for pyrin-inflammasome assembly (2). Such an abnormality results in excessive activation of caspase-1 and increased release of interleukin (IL)-1β and IL-18 (3). Despite its clear genetic origin, FMF exhibits broadly variable phenotypes, severity, responsiveness to the canonical therapies like colchicine, as well as risk of systemic complications, like AA-amyloidosis, which cannot fully be explained by assessment of *MEFV* genotype alone. This variability underscores the potential contribution of different yet unraveled epigenetic and environmental modulators, among which the gut microbiome has emerged to influence symptom presentation and overall quality of life for affected individuals (4, 5). Indeed, the microbiota residing in our gut plays an active role in shaping host immunity, maintaining epithelial barrier integrity, regulating metabolic homeostasis, and modulating a host of complex inflammatory proceedings (6).

Recent insights from gut microbiota research have highlighted how specific microbial composition patterns are recognized as metabolically and immunologically active ‘organs’, composed of trillions of microorganisms that closely interact with the host to regulate digestion, immune maturation, epithelial barrier integrity, pathogen resistance, and cytokine signaling. A balanced microbial ecosystem characterized by beneficial taxa such as *Bifidobacterium*, *Lactobacillus*, *Faecalibacterium prausnitzii*, *Akkermansia muciniphila* and various butyrate-producing *Firmicutes* contributes to immune tolerance and anti-inflammatory homeostasis through the production of short-chain fatty acids (SCFAs). Any alteration of this ecosystem may favor a chronic inflammatory status by increasing exposure to pro-inflammatory microbial antigens and expanding pathobionts, including members of the *Enterobacteriaceae* family, which lead to increased levels of lipopolysaccharides, other toxins, and damage-associated antigens translocating across a weakened epithelial barrier and driving systemic immune activation (7). Indeed, the pyrin-inflammasome has evolved as an innate immune sensor to detect bacterial toxin-induced inactivation of Rho GTPases (8). Microbial-derived toxins and metabolites may therefore act as environmental triggers for FMF flares in genetically predisposed individuals, and SCFAs produced by microbial fermentation of dietary fibers can engage immune regulatory pathways, reinforcing epithelial barrier function and diversifying pro- or anti-inflammatory cytokine production.

The objective of our research was to review the current medical literature correlating gut microbiota with FMF expression, specifically to compare pediatric and adult sceneries, exploring its role on several disease aspects as severity and responsiveness to colchicine administration.

## 2 Methods

**Figure 1:**
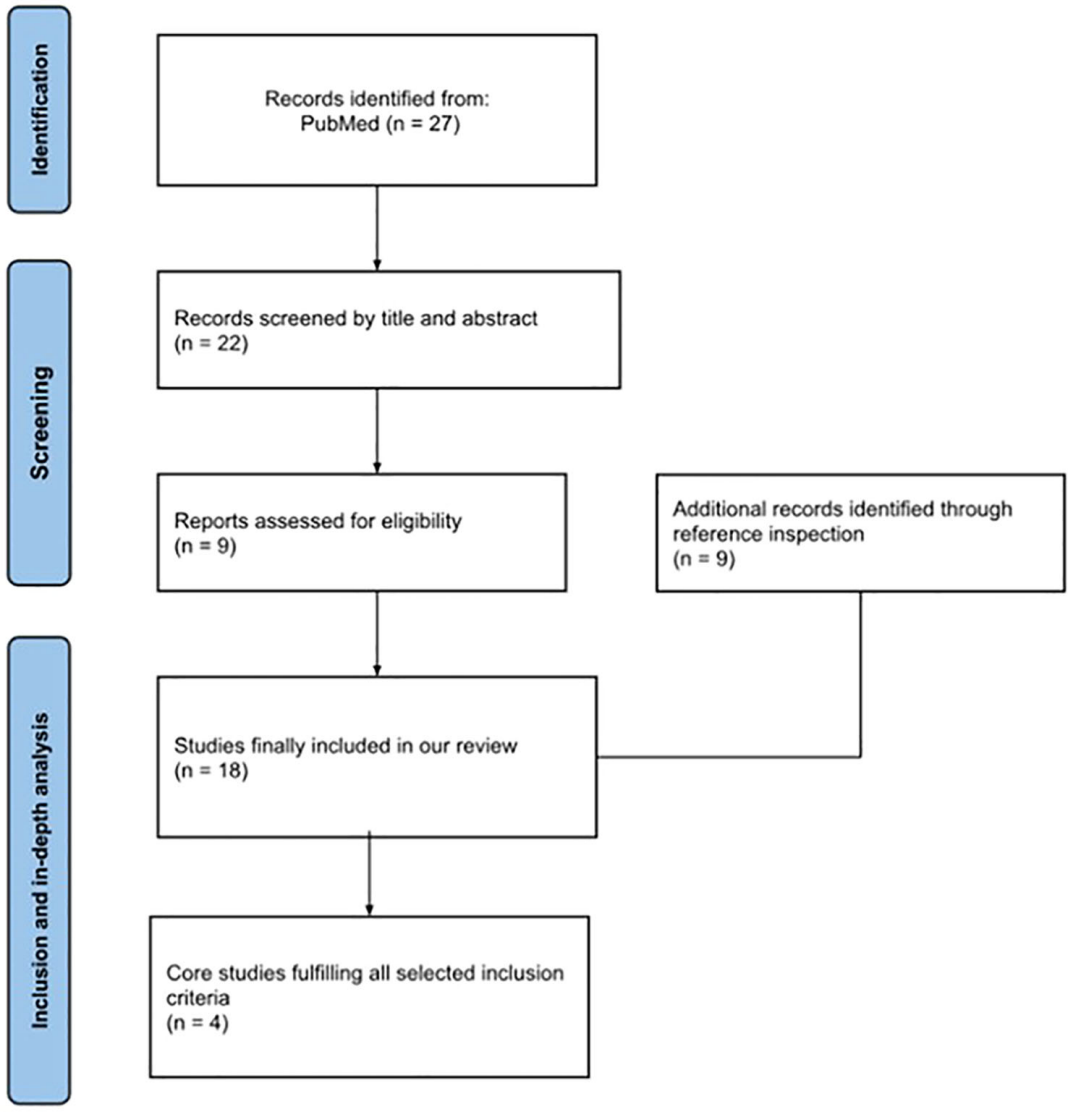
Description of the selection process recruiting the publications dedicated to microbiota and familial Mediterranean fever (FMF) in childhood.

We conducted our comprehensive research without date restrictions on the PubMed library to assess any scientific reports correlating intestinal flora and FMF; the keywords used were “microbiota”, “microbiome”, “intestinal flora” and “FMF”; the literature review was performed until February 2026. Only peer-reviewed articles written in English were included; additional reports were identified and considered through references of retrieved papers with the aim of assessing pediatric data; we also used age filters to focus and extrapolate papers inherent to an age interval ranging from 0 to 18 years. The description of the whole selection process recruiting these publications has been summarized in **Figure 1**. More precisely, full text screening of 27 eligible articles was initially done using Rayyan (RayyanSystems Inc., https://rayyan.ai); all studies were reviewed in full; any discrepancies between reviewers were fixed through open discussion and consensus. After excluding off-topic studies or duplicates, a final number of 4 articles was considered adequate for our objective and selected for in-depth analysis. Unfortunately, data related to pediatric cohorts were very limited. Studies were chosen according to predefined qualitative criteria: (i) direct assessment of gut microbiota composition in FMF patients using molecular profiling techniques in either adults or children; (ii) inclusion of well-characterized FMF cohorts with clinical, genetic, or therapeutic stratification; (iii) relevance to key-unresolved questions in the field, such as disease severity, complications, and age-related differences; and (iv) methodological accuracy and interpretability of microbiota-related findings. This targeted selection highlights, in our opinion, the studies that most clearly define the current state of art on intestinal microbiota alterations in FMF.

## 3 Results

The retrieved studies have been summarized in **Table 1**, showing a coherent and integrative overview of the currently available evidence linking gut microbiota, innate immune dysregulation, and FMF. Given the limited number of investigations directly addressing the microbiota in FMF on pediatric populations, our selection strategy prioritized: (a) studies generating primary microbiome data in clinically stratified FMF cohorts, and (b) one study focusing on gender-specific microbiota patterns and efficacy of probiotic *administration* in patients with FMF. This approach has allowed a structured interpretation of how intestinal microbial composition combined with host genetics might encourage a bidirectional dialogue in FMF. A key-contribution deriving from adult studies is that they move beyond the generic concept of “dysbiosis” and provide disease- and phenotype-linked microbial signatures.

**Table 1:** Descriptive characteristics of the studies related to intestinal microbiota in patients with familial Mediterranean fever included in the construction of the review (FMF, familial Mediterranean fever; AA, amyloid-A; SCFAs, short-chain fatty acids; IL-1, interleukin-1; 16S rRNA, 16S ribosomal RNA; *MEFV*, Mediterranean fever gene).

Reference	Study design/population	Main microbiota findings in FMF	Effect of FMF therapies on microbiota	Key-message
Delplanque et al., 2024 (9)	Large observational association study; adult FMF patients *versus* controls; fecal 16S rRNA sequencing with multivariable modeling	Lower microbial diversity and enrichment of pro-inflammatory taxa (e.g. *Enterobacteriaceae*, *Escherichia/Shigella*, *Ruminococcus gnavus* group)	Colchicine associated with partial microbiota remodeling (↑ *Faecalibacterium* and *Roseburia*); resistant patients show altered microbial network structure	Strong evidence that microbiota composition and structure are linked to FMF expression and treatment response
Deshayes et al., 2019 (10)	Cross-sectional observational study; adult FMF patients with and without AA amyloidosis *versus* healthy controls; fecal 16S rRNA sequencing	Reduced microbial α-diversity and distinct fecal microbial profile; enrichment of specific *Clostridiales*	Colchicine does not normalize gut microbiota composition	First demonstration that FMF is associated with a disease-specific dysbiosis, supporting a role for microbiota as a modifier of chronic inflammation and potential risk of complications
Ozen et al., 2021 (11)	Multicenter international pediatric study; children with FMF *versus* healthy controls; fecal 16S rRNA sequencing	Marked geographic differences in the microbiota composition; no consistent FMF-specific dysbiosis and no relationship with disease severity	All pediatric patients were on colchicine; no evidence of microbiota normalization was ascertained	Environmental and geographic factors outweigh disease-related microbiota signatures in children with FMF
Pepoyan et al., 2021 (12)	Observational interventional study (gender-stratified cohort with probiotic intervention)	Taxon-specific microbiota alterations in FMF, particularly involving *Prevotella* spp., with loss of physiological gender-related microbial differences in patients compared with healthy controls	Immunobiotic (*Lactobacillus acidophilus* Narine) associated with partial modulation of *Prevotella* abundance and inflammatory markers, suggesting microbiota-targeted strategies as potential adjunctive tools	Context-dependent framework describing gut microbiota as a dynamic interface between innate immune activation (pyrin–inflammasome pathway), environmental exposure, and inflammatory phenotype expression in FMF

In a quite large French cohort analyzed by Delplanque et al. (119 FMF patients aged 32–59 years *versus* 61 controls) via 16S rRNA sequencing, FMF microbiota differed from controls due to enrichment of taxa implicated in inflammatory states, including *Enterobacter*/*Klebsiella* and the *Ruminococcus gnavus* group. Crucially, the analysis was clinically supported: multivariable models adjusted for relevant confounders (genotype, AA-amyloidosis, bowel habits, colchicine exposure, colchicine resistance, eventual biological therapy) demonstrated that disease characteristics could be mapped onto specific taxa. In particular, severe FMF was associated with the expansion of the *Ruminococcus gnavus* group and *Paracoccus*, while colchicine exposure correlated with expansion of anti-inflammatory taxa such as *Faecalibacterium* and *Roseburia*. Colchicine-resistant patients also showed an altered ecosystem architecture, with decreased inter-taxa connectivity on network analyses, suggesting that treatment failure is not only a matter of which taxa are present but also of loss of microbial community connections (9).

Deshayes et al. highlighted the evidence that dysbiosis in FMF is not a random finding by linking microbiota differences to a major long-term complication. Indeed, in this cross-sectional study performed via 16S rRNA sequencing on Illumina MiSeq, FMF patients (with and without AA-amyloidosis, with an age range of 46-60.5 and 31.5–56 years, respectively) were compared not only with healthy controls but also with patients affected by AA-amyloidosis due to non-FMF causes (having and age range of 47-68.5 years), enabling a separation between “FMF-related signals” and “amyloidosis-related signals”. FMF was associated with a reduction in α-diversity and a significantly altered overall composition (β-diversity differences *versus* controls). Within FMF, AA amyloidosis was associated with additional microbiota shifts (including Operational Taxonomic Units within *Clostridiales*) alongside distinct inflammatory/metabolic signals (e.g., higher adiponectin and increased indoleamine 2,3-dioxygenase activity), supporting the interpretation that microbiota may be involved not only in the “baseline” FMF inflammation”, but also in the biology of complications (10). Together, these adult datasets converge on a clinically meaningful model: FMF is associated with ecosystem perturbations in terms of diversity or composition and taxonomic shifts, favoring inflammation-associated groups (e.g., *Enterobacteriaceae* members and *Ruminococcus gnavus*), with differences relatable to phenotype severity, complications (as AA-amyloidosis), or response to treatment. Importantly, the Delplanque study also provided a direct evidence, within FMF cohorts, that colchicine may have a microbiome-related footprint: exposure is associated with expansion of taxa widely considered anti-inflammatory (*Faecalibacterium*, *Roseburia*) and reduction of taxa enriched in the most severe disease expression (*Ruminococcus gnavus* group, *Paracoccus*), suggesting microbiome modulation rather than normalization as a plausible component of the therapeutic effect by colchicine. The practical implication is that stratifying patients by *MEFV* genotype alone is insufficient, requiring that future precision models of disease management should incorporate intestinal microbiota/ecosystem features (10).

The pediatric perspective, captured by Ozen et al., was essential to the aim of this review because it may prevent any overgeneralization deriving from adult cohorts of FMF patients and clarified what is currently *not* demonstrated in childhood. This international pediatric study compared children with FMF and controls across two geographic settings (Turkey and United States of America) using the 16S rRNA profiling and assessing disease clinical severity. The central result was negative, but informative: within each country, α- and β-diversity did not differ significantly between FMF and controls, and microbial community composition did not predict disease severity. At the same time, the study documented strong geography-driven microbiota differences: Turkish cohorts (mean age: 11 years) showed higher relative abundance of *Bacteroidia*, while American cohorts (mean age: 8.5 years) showed higher rates of *Chlostridia*, highlighting how peculiar environmental contexts can influence or even dominate microbial variations in children and potentially mask subtle disease-related signals. Methodological constraints (small sample size, limited controls, and processing in two laboratories despite similar protocols) were explicitly acknowledged by the authors, helping to elucidate the absence of a robust FMF-specific signature in childhood as not currently detectable, rather than a definite evidence of absence. In fact, gut microbiome composition may differ across regions and ethnicities, and changes over time can be influenced by multiple factors, including diet, lifestyle, hormonal cycles, comorbidities, and exposure to different antimicrobial agents (11).

A complementary perspective was offered by the Armenian study by Pepoyan et al., who focused on gender-specific microbiota patterns and the effect of the immunobiotic *Lactobacillus acidophilus* Narine in 24 male and 24 female FMF volunteers (age range of participants: 18–50 years, all patients were from Yerevan, Armenia). This study identified taxon-specific shifts, particularly involving *Prevotella* spp., and demonstrated that physiological sex-dependent differences in *Prevotella* abundance observed in healthy controls were lost in FMF patients, suggesting that chronic inflammatory activity may disrupt baseline microbial variability. The authors also discussed the potential relevance of Gram-negative-derived lipopolysaccharides in sustaining innate immune activation in genetically predisposed individuals, reinforcing the concept of a bidirectional interaction between microbial composition and pyrin-inflammasome driven inflammation. Furthermore, probiotic intervention was associated with partial modulation of *Prevotella* abundance and inflammatory markers, supporting the hypothesis that targeted microbiota modulation could influence inflammatory level. This study is particularly relevant for our investigation of the microbiota-FMF axis because it moves beyond the descriptive identification of dysbiosis and addresses three key-questions central to the field. First, it supports the concept that microbial alterations in FMF are not uniform but context-dependent, influenced by host-related variables such as sex and environmental background. Second, it provides preliminary evidence that microbiota composition in FMF is biologically responsive and modifiable, suggesting that microbial shifts are not merely epiphenomena of inflammation but part of a dynamic host-microbe equilibrium. Third, by linking specific taxa to inflammatory markers and discussing microbial-derived products as potential drivers of innate immune activation, it strengthens the plausibility of a mechanistic bridge between microbiota composition and pyrin-inflammasome activity. Furthermore, this study shifts the focus from the search for a universal “FMF dysbiotic signature” toward a different interpretation in which disease expression may depend on context-specific microbial configurations. By highlighting gender effects, regional background, and probiotic responsiveness, Pepoyan et al. highlighted the heterogeneity of microbiota patterns within FMF and suggested that microbial alterations may reflect dynamic host-environment interactions rather than a fixed disease marker (12).

## 4 Discussion

Overall, the selection of these studies reflects an effort to balance direct microbiota evidence and clinical relevance. Adult cohorts provide convergent signals of FMF-associated dysbiosis linked to phenotype, complications, and treatment response, whereas the pediatric dataset delineates the current limits of detectability of a stable disease-specific signature in childhood and underscores the weight of geography and environmental exposures. The Armenian experience further refines this framework by demonstrating that microbiota patterns in FMF are not only age-dependent but also gender- and context-sensitive. This observation therefore supports integrating apparently-divergent results into an age- and context-dependent model in which microbiota changes may emerge, fluctuate, and potentially stabilize over time under the combined influence of genotype, recurrent inflammation, environmental exposures, and FMF-directed therapies. This integrated analysis supports a model in which FMF arises from the dynamic interplay between host genetics, innate immunity, environmental factors, and gut microbial ecology, providing an interesting structured and biologically plausible framework for future research.

Several studies have documented compositional shifts in the gut microbiota of FMF patients compared with healthy individuals. Dysbiosis may decrease intestinal barrier integrity, increase permeability and facilitate translocation of microbial products, which in turn activate peripheral immune responses. Distinct differences between FMF and healthy microbiota are evident at both phylum and family levels. For example, an increase in several pro-inflammatory taxa has been observed, including members of the *Proteobacteria* family (such as *Enterobacter* and *Klebsiella*) as well as taxa belonging to the *Ruminococcus gnavus* group (9). Microbiome alterations are also related to some clinical phenotypes: those displaying severe clinical manifestations and colchicine resistance or those having homozygous *MEFV* mutations exhibited a microbial signature characterized by increased abundance of *Escherichia* and *Shigella* (9). The medical literature shows that specific changes in fecal microbiota of FMF patients, including reduced microbial diversity, depletion of butyrate-producing taxa and enrichment of pro-inflammatory bacteria, were associated both with FMF and FMF-related amyloidosis, suggesting a pathogenic role of intestinal microorganisms and a potential therapeutic target (10). In a study evaluating acute attacks of FMF patients, a less diverse and depleted gut microbiota was reported in comparison with healthy controls, with reduction of *Prevotellaceae*, *Dialister* and *Prevotella*, alongside increased levels of *Porphyromonadaceae*, *Parabacteroides* and *Faecalibacterium* (10). Pediatric studies found geographic differences in the gut microbiota composition between FMF patients living in Turkey and those living in the United States of America, highlighting the influence of environmental and lifestyle factors on disease severity (11).

Colchicine is the first-line therapy in all FMF patients (13). Patients with unclassified inflammatory bowel disease carrying *MEFV* mutations may even respond well to colchicine treatment (14). Conversely, patients who respond to colchicine exhibit higher inter-taxa connectivity and increased abundance of anti-inflammatory genera such as *Faecalibacterium* and *Roseburia* (9). Long-term colchicine treatment does not normalize FMF-associated microbiota alterations, but induces a distinct and stable remodeling of microbial-derived metabolites, characterized by the formation of LCFA profiles that are clearly separable from both untreated FMF patients and healthy controls (15). Biologic agents have been used in colchicine-resistant FMF patients or in patients with comorbidities who are intolerant to colchicine; however, factors influencing the initiation of biological treatment as gender, delay in diagnosis, family history of FMF, amyloidosis or homozygous exon 10 and compound heterozygous exon 10 *MEFV* mutations are not known (16, 17). Evidence directly addressing the relationship between biologic therapies, gut microbiota, and FMF is currently limited. However, increasing data from other chronic inflammatory and autoinflammatory conditions such as inflammatory bowel diseases indicate that biologic agents, particularly IL-1 inhibitors, may indirectly influence gut microbiota composition and function by modulating systemic inflammation, intestinal permeability, and mucosal immune responses, raising the hypothesis that gut microbiota profiles could modulate therapeutic efficacy in patients receiving biologic agents. Indeed, a depletion of core gut microbes and expansion of bacteria typical of the oral cavity have been associated with baseline disease severity in patients with ulcerative colitis, while remission has been linked with species-specific temporal changes that may be implicative of therapy efficacy (18). Future longitudinal studies integrating microbiome profiling, immunophenotyping, and clinical outcomes are needed to clarify the bidirectional interplay between biologic therapies and gut microbiota in FMF, and future microbiome engineering might be a useful approach to control FMF and other rheumatic diseases, for example, via correction of the altered signaling pathways, production of metabolites with drug-like activities or anti-inflammatory molecules. Nutritional interventions, including specific diets, probiotics, and micronutrient supplementation, have not been explored as potential strategies to modulate FMF symptoms, and from a clinical perspective, there is currently insufficient evidence to recommend microbiome-based or dietary interventions as standard treatment. Nevertheless, individualized nutritional counseling may be considered within a multidisciplinary framework, and future research should focus on well-designed, large-scale studies that critically assess the role of gut microbiome alterations and nutritional strategies in FMF.

## 5 Conclusions

The relationship between gut microbiome dysbiosis, nutrition and FMF is an emerging relevant field of interest, but currently remains largely speculative, mostly in pediatrics. The clinical significance of the currently available evidence related to adult patients is preliminary and often lacks replication in larger independent cohorts, then it should not be overestimated, though a physiological crosstalk between gut microbiota and innate immunity could modulate FMF expression and severity, highlighting this axis as a fertile area for mechanistic investigation and therapeutic innovation. A growing number of studies suggests that human gut microbiota plays a previously underappreciated role in influencing inflammatory features of FMF. Intestinal microbiota alteration in FMF resembles microbial patterns observed in other inflammatory disorders and aligns with hypersensitivity of FMF immune cells to microbial-derived stimuli. The mutual interaction between microbiota and pyrin-inflammasome reveals that microbial-derived toxins are capable of activating inflammatory pathways in FMF, helping to explain why FMF-associated dysbiosis appears relatively stable across patients and why microbial profiles correlate with genotype, disease severity, and treatment responsiveness. These insights also carry therapeutic implications, as colchicine which is the cornerstone of FMF management, has been associated with an alteration of the gut microbial metabolomic and taxonomic profile, suggesting that part of colchicine efficacy may involve microbiome modulation rather than its complete normalization. Future research should integrate high-resolution microbiome profiling with immunophenotyping and genotype analysis to better characterize the interplay between host microbiota and innate immunity in patients with FMF and ideally find more personalized treatments.
